# *Clostridioides difficile* and Gut Microbiota: From Colonization to Infection and Treatment

**DOI:** 10.3390/pathogens13080646

**Published:** 2024-07-31

**Authors:** Patrizia Spigaglia

**Affiliations:** Department of Infectious Diseases, Istituto Superiore di Sanità, 00161 Roma, Italy; patrizia.spigaglia@iss.it; Tel.: +39-06-4990-2822

**Keywords:** *Clostridioides difficile*, CDI, PCR-ribotype, colonization, gut microbiota, AAD, FMT, probiotics, prebiotics, postbiotics

## Abstract

*Clostridioides difficile* is the main causative agent of antibiotic-associated diarrhea (AAD) in hospitals in the developed world. Both infected patients and asymptomatic colonized individuals represent important transmission sources of *C. difficile*. *C. difficile* infection (CDI) shows a large range of symptoms, from mild diarrhea to severe manifestations such as pseudomembranous colitis. Epidemiological changes in CDIs have been observed in the last two decades, with the emergence of highly virulent types and more numerous and severe CDI cases in the community. *C. difficile* interacts with the gut microbiota throughout its entire life cycle, and the *C. difficile*’s role as colonizer or invader largely depends on alterations in the gut microbiota, which *C. difficile* itself can promote and maintain. The restoration of the gut microbiota to a healthy state is considered potentially effective for the prevention and treatment of CDI. Besides a fecal microbiota transplantation (FMT), many other approaches to re-establishing intestinal eubiosis are currently under investigation. This review aims to explore current data on *C. difficile* and gut microbiota changes in colonized individuals and infected patients with a consideration of the recent emergence of highly virulent *C. difficile* types, with an overview of the microbial interventions used to restore the human gut microbiota.

## 1. Introduction

*Clostridioides difficile* is an anaerobic, Gram-positive, and endospore-forming bacillus, known as the most important and common cause of nosocomial antibiotic-associated diarrhea (AAD) worldwide [1].

*C. difficile* spores can survive in environments for years. After the oral ingestion of spores from infected individuals or the environment, *C. difficile* can transitorily or permanently colonize the human intestine, especially in infants and hospitalized patients [2]. Asymptomatic individuals colonized by toxigenic *C. difficile* strains have received growing interest as a potential transmission source of this pathogen. In fact, *C. difficile* carriers have been associated with significant skin and environmental contamination, similarly to patients with a *C. difficile* infection (CDI) [3].

CDI presents a large range of symptoms, from mild diarrhea to severe manifestations such as pseudomembranous colitis and toxin megacolon, which can lead to the death of patients [4]. Only toxigenic *C. difficile* strains cause infection and these usually produce two toxins, toxin A (TcdA) and toxin B (TcdB), that are the main virulence factors of this pathogen [5]. The *tcdA* and the *tcdB* genes are located in a chromosomal element, the pathogenicity locus (PaLoc), which also harbors regulatory genes for these toxins’ expression and release into the extracellular environment [5]. Furthermore, *C. difficile* strains can also produce a third toxin, known as the binary toxin CDT, which enhances their virulence and adherence to intestinal cells [6]. The production of this toxin characterizes several *C. difficile* types, such as the PCR-ribotype (RT) 027 and the RT 078. These types are characterized by high virulence and a resistance to antibiotics, and they have been associated with severe infections, an increased frequency of CDI recurrences, and higher mortality rates [7,8].

The human gut microbiota is a complex ecosystem that has a crucial role in conferring host immunity and preventing colonization and invasion by pathogens [9]. The gut microbiota is composed of an incredible number of microorganisms that can vary between individuals and that can be altered by various factors [10]. Dysbiosis is defined as an alteration in the gut microbiota’s composition, metabolic activities, or distribution [11]. The recent advances in high-throughput sequencing, mass spectrometry, and other omics approaches have improved the volume of data available on the composition of the gut microbiota, facilitating our understanding of the causes and possible impacts of dysbiosis [12,13].

*C. difficile* interacts with the gut microbiota throughout its entire life cycle. *C. difficile*’s role as a colonizer or invader largely depends on changes in the gut microbiota’s components, which *C. difficile* itself can promote and maintain [14]. Antibiotic exposure is considered the primary risk for CDI. Gut microbiota perturbations due to antibiotic administration allow *C. difficile* spores to germinate in vegetative forms that can colonize the intestine and produce toxins, leading to the inflammation of the intestinal membrane and subsequent symptoms of CDI [15,16,17]. 

The restoration of intestinal homeostasis has emerged as a promising therapeutic approach for CDI. In particular, fecal microbiota transplantation (FMT), a method used to normalize the composition of the gut microbiota of a recipient via the transplantation of a small sample of feces from a healthy donor, is widely used as a therapeutic option for recurrent CDIs [18,19]. However, alternative strategies to re-establish intestinal eubiosis for the prevention and treatment of CDI are under investigation [20,21,22].

This review aims to explore the current data on *C. difficile* and gut microbiota changes in colonized individuals and infected patients, with a consideration of the recent emergence of highly virulent *C. difficile* types, and to provide an overview of the microbial interventions used to restore the human gut microbiota in patients with CDI.

## 2. Healthy Gut Microbiota

The human intestinal microbiota is a dynamic functional interface between the external environment and the human body, and its composition increases in microbial diversity in the childhood, reaching stability during adulthood [23].

The components of the gut microbiota include several species of bacteria, yeasts, archaea, and viruses. The dominant bacterial phyla are Firmicutes, Bacteroidetes, Actinobacteria, Proteobacteria, Fusobacteria, and Verrucomicrobia, with a high prevalence of Firmicutes and Bacteroidetes (90%) [24,25]. The Firmicutes phylum includes more than 200 different genera, with the *Clostridium* genera being prevalent (95%), while Bacteroidetes includes two predominant genera, *Bacteroides* and *Prevotella* [24].

The different regions of the gastrointestinal tract are colonized by different species, with most of the microorganisms present in the colon. In the human large intestine, the genus *Bacteroides* and two families belonging to the Firmicutes phylum, *Lachnospiraceae* and *Ruminococcaceae*, are prevalent (50–70%), while, in the small intestine, the Firmicutes and the Actinobacteria, the latter mainly represented by the *Bifidobacterium* genus, are the prevalent phyla [26].

The gut microbiota’s composition changes with the age. For example, while the intestinal community of infants is rich in *Bifidobacterium* spp., in elderly individuals a decreased proportion of *Bifidobacterium* spp., *Lactobacillus*, and the *Faecalibacterium* and *Ruminococcaceae* families have been observed, with an increased abundance of Proteobacteria, Bacteroidetes, and *Clostridium* spp. [24,27,28].

Although the composition of the gut microbiota is different in different individuals, characterized by specific bacterial strains [29], a classification based on its taxonomic composition proposes three different enterotypes: enterotype I, more frequently enriched in *Bacteroides*; enterotype II, more frequently enriched in *Prevotella*; and enterotype III, more frequently enriched in *Ruminococcus* [30]. However, there are controversial opinions on this classification, which is not accepted by the entire scientific community [31,32].

## 3. *C. difficile* Colonization Resistance

**Figure 1:**
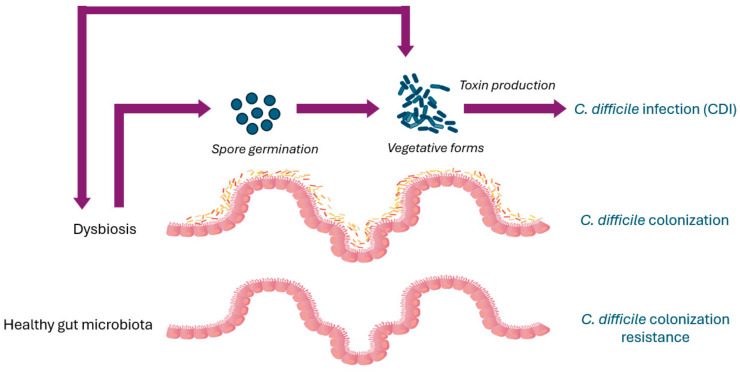
*Clostridioides difficile* pathogenesis and gut microbiota.

The components of a healthy gut microbiota form a stable community and their biochemical activities have a beneficial impact on the host, conferring resistance to non-native bacteria, developing the host immune system, and exerting important metabolic functions [33,34]. The capability of the gut microbiota to prevent colonization by pathogens, including *C. difficile*, is known as colonization resistance (Figure 1). Different mechanisms are involved in the colonization resistance against *C. difficile*, such as the production of bile acids and metabolites, competition for nutrients, and the stimulation of the immune response of the host [35]. The administration of antibiotics has been demonstrated to cause a shift of the bile acid pool, with a consequent increase in *C. difficile* spore germination [36,37]. Furthermore, the reduction in bacteria with a bile-metabolizing capacity results in the enrichment of primary bile acids, which promotes the germination of *C. difficile* spores, and in the loss of secondary bile acids, which inhibit *C. difficile* growth [38,39,40,41]. Among the bacteria with a bile-metabolizing capacity, *Clostridium scindens* has been associated with *C. difficile* colonization resistance in a gnotobiotic mouse model [38,42], while *Clostridium butyricum* and *Roseburia intestinalis*, both butyrate producers, are strongly associated with CDI-negative samples [43].

Metabolites produced by the gut microbiota (or metabolome), such as sugars, amino acids, and lipids, may also have an impact on *C. difficile* colonization resistance [44,45]. For example, short-chain fatty acids (SCFAs), such as butyrate, have been associated with colonization resistance against *C. difficile,* since they are able to inhibit the growth of this pathogen and to affect the immunological response of the intestinal mucosa in vitro [44,46]. Intestinal microbiota components also produce bacteriocins, which are antimicrobial peptides with a narrow or broad spectrum of activity against other bacteria and pathogens [47]. Bacteriocins produced by strains of *Bacillus*, *Lactococcus,* and *Enterococcus* have been demonstrated to exert an antimicrobial effect against *C. difficile in vitro* [48,49,50].

The intestinal microbiota’s components can also directly compete with *C. difficile* through the consumption of nutrients; in particular, bacteria with similar nutritional requirements can protect the host. Interestingly, it has been observed that colonization by a nontoxigenic strain of *C. difficile* protects against colonization by toxigenic *C. difficile* in murine models [51].

Interestingly, the gut microbiota interacts with the innate and adaptive immune system of the host and modulates its activity to maintain homeostasis and inhibit inflammation via metabolic products that interact with and regulate immune cells [52,53]. The stimulation of local innate lymphoid cells ILC1s by microbiota components has been found to be implicated in the protection against *C. difficile* through interferon (IFN)-γ production [34]. Among the gut microbiota’s components, the commensal *Clostridia* can promote the development of intraepithelial lymphocytes (IELs), particularly alpha beta T-cell receptor (TcR)-bearing T cells and immunoglobulin A (IgA)-producing cells, through a gradient of SCFAs and secondary bile acids, contributing to the maintenance of overall gut function [54,55]. Several authors have reported that *C. butryicum* promotes resistance to CDI through metabolic and immune modulation, suggesting that this bacterium might be potentially used as a treatment for CDI [56,57,58,59]. Finally, the products of some metabolic pathways related to tryptophan or polyamines biosynthesis metabolism, which have an anti-inflammatory effect, can contribute to the protection against CDI and to the restoration of gut homeostasis [60,61,62,63].

## 4. Asymptomatic *C. difficile* Colonization

*C. difficile* colonization is usually defined as the detection of *C. difficile* or its toxins in the absence of diarrhea or without findings consistent with pseudomembranous colitis [2,64].

Unusually high levels of *C. difficile* asymptomatic colonization have been reported in neonates and infants, with rates up to 90% and levels of bacterial burdens similar to those found in adults with CDI [65]. *C. difficile* rates of colonization remain high (22%) up to 2 years of age, declining to 1–3% with the maturation of both the gut microbiota and the immune system of the host [66].

Among the possible factors protecting infants from CDI, it has been hypothesized that the lack of the receptors for *C. difficile* toxins in the intestine cells and the acquisition of maternal antibodies via breastmilk could have a role [67]. Also, it has been hypothesized that breastfed babies may be more protected from colonization by *C. difficile* due to a reduced mean fecal pH (5.29 vs. pH 6.48 of bottle-fed infants), possibly related to the reduced buffering capacity of breastmilk [68].

Relatively low percentages of asymptomatic *C. difficile* colonization have been reported in adults, although these percentages can dramatically increase in individuals who have contact with healthcare structures, particularly the elderly, with rates up to 51% [17,69]. It has been hypothesized that the development of antibodies may be one of the mechanisms involved in *C. difficile*’s asymptomatic colonization due to the very high levels of antibodies against the TcdA observed in *C. difficile* carriers [70]. Antibodies against *C. difficile* toxins may contribute to CDI protection directly, by a toxin neutralizing effect, and indirectly, by limiting epithelial damage and restoring colonization resistance [71,72,73].

Asymptomatic *C. difficile* carriers are usually undetected due to a lack of routine screening, but there has been increased attention given to them as a reservoir of this pathogen in both healthcare facilities and in the community. The importance of asymptomatic *C. difficile* carriers in hospital wards has been highlighted in a recent study, showing that 2.6% of patients not exposed to asymptomatic carriers develop CDI, while this percentage rises to 4.6% when the patients are exposed to carriers [74]. Another study using a mathematical model to analyze the transmission of *C. difficile* in hospital wards has demonstrated that the contribution of colonized patients at admission and patients with CDI to new colonizations are similar [75]. *C. difficile* carriers also have an important role in the community; in fact, a recent study estimating the risks of community-associated CDI (CA-CDI) has reported increased CDI rates among residents ≥ 65 years old in comparison to controls residing at home [76]. Furthermore, close contact with infants ≤ 2 years, frequently colonized by *C. difficile*, has been identified as a potential risk factor for CA-CDI in a prospective case–control study in the UK [77].

Finally, individuals colonized by toxigenic strains have a higher risk of developing CDI compared to non-colonized individuals; therefore, colonization with *C. difficile* is considered a crucial factor in the progression to CDI [78,79].

## 5. Gut Microbiota in Asymptomatic *C. difficile* Carriers

Age, as well as other factors, such as the use of antibiotics, proton pump inhibitors (PPIs), and hospitalization, may affect the gut microbiota and predispose a person to *C. difficile* colonization (Figure 1). For this reason, several studies have investigated the alterations in the gut community structure of neonates/infants and adults colonized by *C. difficile*, compared to healthy individuals and patients with CDI, providing important information on the intestinal microbiota’s components in relation to the age.

### 5.1. Neonates and Infants

**Figure 2:**
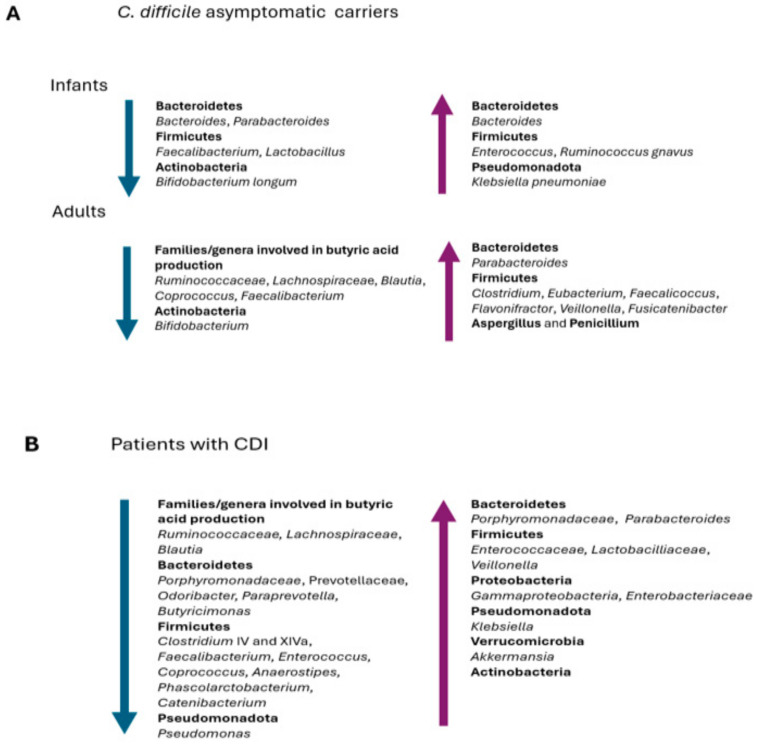
Prevalent changes observed in the gut microbiota of (**A**) infants and adults asymptomatic *C. difficile* carriers and (**B**) patients with CDI.

The high rates of *C. difficile* colonization observed in infants suggest that the microbiota in the early life is a favorable environment for this pathogen. Interestingly, many bacteria that antagonize *C. difficile*, such as *Lactobacillus* species, have been negatively associated with *C. difficile* colonization in neonates (Figure 2A). In fact, it is known that *Lactobacillus gasseri* and *Lactobacillus acidophilus* reduce the *C. difficile* burden and inhibit the expression of the TcdA, respectively [67]. In infants, *C. difficile* colonization has also been negatively associated with *Bifidobacterium longum*, which is able to inhibit *C. difficile* growth and toxin production by decreasing the intestinal pH, while *Ruminococcus gnavus* and *Klebsiella pneumoniae* are more frequently detected in the microbiota of colonized infants [67,80,81,82] (Figure 2A). Antibiotic treatment has been associated with a decrease in microbial diversity and often with an outgrowth of *Enterobacteriaceae* [83]. Interestingly, infants born via cesarean section have been found to be enriched in *Enterococcus*, which is associated with increased rates of *C. difficile* colonization [67].

Different authors have observed a correlation between an increase in specific gut anaerobes and a reduction in *C. difficile* colonization in infants and children [67,80,84,85]. Although a *Bacteroides* enrichment has been found in neonates with vaginal delivery and an exclusively breastmilk diet, which are negatively associated with *C. difficile* colonization, it is still unclear whether *Bacteroides* may have a direct or indirect effect on CDI protection in early infancy [67,86].

The analysis of the microbiota composition of an infant colonized by *C. difficile* has shown important changes during weaning, with an increase in the relative abundance of *Bacteroides* spp., *Blautia* spp., *Parabacteroides* spp., *Coprococcus* spp., *Ruminococcus* spp., and *Oscillospira* spp., suggesting that the increased level of some bacterial groups during weaning can be associated with a reduction in *C. difficile* colonization [87].

Although further studies are necessary to better understand *C. difficile* colonization as a potential protection from developing CDI, some data support the fact that neonates and infants might potentially benefit from asymptomatic *C. difficile* colonization. In particular, the IgA and IgG responses to *C. difficile* toxins might be protective against CDI in infants colonized by toxigenic *C. difficile* strains and, also, in breastfed infants [4,65].

### 5.2. Adults

A lower microbial diversity and species richness has been reported in the gut microbiota of adults colonized by *C. difficile* compared to healthy subjects [88]. A depletion or lack of butyrate-producing bacteria, such as *Ruminococcaceae* (*Faecalibacterium* and *Ruminococcus)*, *Lachnospiraceae* (*Blautia* and *Coprococcus)*, and *Bifidobacterium*, has been reported by Vázquez-Cuestain et al. in *C. difficile* carriers [89] (Figure 2A). The authors further identified the genera *Parabacteroides*, *Faecalicoccus*, *Flavonifractor*, and *Clostridium*_XVIII as biomarkers for patients colonized by *C. difficile* (Figure 2A).

Other authors have also found that the gut microbiota of asymptomatic carriers exhibits a relative abundance of *Clostridium* and *Eubacterium* compared to patients with CDI, and that *Veillonella*, *Fusicatenibacter,* and *Eubacterium* species may play a role in preventing CDI in asymptomatic *C. difficile* carriers [90,91].

Finally, a study has recently demonstrated that the gut microbiota of *C. difficile*-colonized patients shows a higher relative abundance of the genera *Aspergillus* and *Penicillium* compared to patients with CDI and healthy controls, suggesting that indirect interactions between fungi and bacteria may have some effects on *C. difficile* and on treatment effectiveness [92] (Figure 2A).

All these data indicate a decrease in species richness/diversity in the gut microbiota of asymptomatic *C. difficile* carriers and that the presence of certain bacterial groups might be protective, preventing *C. difficile* overgrowth or the transition from asymptomatic colonization to infection.

## 6. Gut Microbiota Dysbiosis Increases Susceptibility to *C. difficile* Infection

The composition and function of the gut microbiota are influenced by exposure to a changing environment and factors such as antibiotics, age, medical treatments, diet, etc. [24,93]. The imbalance of the gut microbiota ecosystem, or dysbiosis, determines a shift in the composition and/or function of bacterial components, which can lead to increased CDI susceptibility [38,94,95,96] (Figure 1).

The excessive/wrong use of antibiotics, particularly those with a broad spectrum of action, represents the main risk for CDI, reducing the gut microbiota’s diversity and increasing gut colonization [97,98,99]. Gut microbiota changes due to an exposure to antibiotics can persist for a long time. In fact, treatment with ciprofloxacin, clindamycin, clarithromycin, or metronidazole has been demonstrated to cause alterations in the gut microbiome lasting up to four years [100,101,102]. Furthermore, antibiotics can affect the metabolic function of the endogenous microbiota. For example, *Bacteroides* spp. produce succinate and liberate sialic acid and fucose, promoting *C. difficile* colonization, only when the host glycans became available in the gut after antibiotic exposure [103,104]. Changes in the components of the gut microbiota also depend on the different classes of antibiotics used [105]. A study on the effects of antibiotics associated with CDIs in a mouse model has demonstrated that the usage of clindamycin determines a decrease in obligate anaerobic bacteria and a prevalence of Enterobacteriaceae, while a prevalence of *Pseudomonadaceae* and *Lactobacillaceae* has been observed after cephalosporin exposure, and a lower prevalence of Bacteroidetes and a higher prevalence of Proteobacteria when tigecycline is used [106]. Furthermore, a decrease in Bacteroidetes and *Bifidobacteria* has been observed using a cefotaxime instillation in a human gut model, with subsequent *C. difficile* germination and the production of high levels of toxins [107].

Interestingly, the target population of CDIs is represented by patients ≥ 65 years old, and increasing age may be implicated in intestinal microbiota changes. In fact, lower microbial diversity and an altered microbiota composition have been observed in elderly people, with a decrease in protective bacteria such as *Bifidobacteria*, Firmicutes, and Bacteroidetes and an increment in those associated with disease, such as Proteobacteria [108]. In addition, the more frequent use of antibiotics, frequent hospitalizations, and the comorbid conditions that commonly occur in the elderly contribute to their decreased resistance to *C. difficile*’s colonization and infection [4].

The use of PPIs may also have an impact on the gut microbiota. Studies in vitro have demonstrated that PPIs cause a decrease in the relative abundance of Bacteroidetes and an increase in Firmicutes, particularly *Lactobacillus*, which may increase susceptibility to *C. difficile* [109,110].

Furthermore, obesity, autoimmune and allergic diseases, diabetes, and inflammatory bowel disease may have a role in gut microbiota perturbations [111]. Interestingly, an interaction between the gut microbiota and obesity has been reported in humans, resulting in a decrease in Bacteroidetes and an increase in Firmicutes, although a clear trend between this ratio and obesity status has not been demonstrated [112,113].

## 7. *C. difficile* Infection Pathogenesis

*C. difficile* transmission occurs via the fecal–oral route, with the ingestion of either vegetative forms or spores. *C. difficile* spores are metabolically inert, extremely resilient, and resistant to oxygen, so they can survive for long periods in the environment [114]. *C. difficile* germination initiates in response to chemical signals, such as bile acid taurocholate, that indicate that environmental conditions may be conducive to the survival of the vegetative cells and their growth [115]. After the induction of the spores’ germination and the replication of vegetative cells, the production of toxins leads to the symptomatic disease (Figure 1).

The major virulence factors produced by *C. difficile* are the enterotoxin TcdA and the cytotoxin TcdB [116]. The genes encoding for these toxins and for the accessory proteins TcdR, TcdC, and TcdE, involved in the regulation of toxin production and in their extracellular extrusion, are located on a chromosomal element of 19.6 kb, known as the pathogenicity locus (PaLoc) [117,118]. TcdA and TcdB show approximately 66% amino acid sequence similarity, and both present two conserved domains, a catalytic glucosyltransferase domain and a cysteine protease domain, necessary for the auto-processing and delivery of the catalytic domain to the cytosol of the intoxicated cells [6]. In the cytosol, the catalytic domain inactivates several cellular proteins belonging to the RAS homolog (Rho) family that catalyze the hydrolysis of guanosine triphosphate (GTPases), which are crucial in the regulation of the actin cytoskeleton, leading to alterations in cellular integrity and morphology, a loss of the tight junctions, and the increased permeability of the intestine epithelial layer [118,119]. Although TcdA and TcdB act synergistically, strains with a TcdA^−^/TcdB^+^ phenotype may cause severe infections and even mortality [120].

In addition to TcdA and TcdB, several *C. difficile* types, prevalently those which are highly virulent, also produce an additional toxin, the binary toxin CDT, which has been associated with more severe diseases, an elevated risk of recurrence, and high mortality rates [121,122]. CDT belongs to the family of *Clostridial* iota-like toxins and shows two components, the enzymatic component CdtA and the binding component CdtB, encoded by genes located on a 6.2 kb region designated as the Cdtlocus (CdtLoc) [123]. The internalization of the enzymatic component CdtA into the cytosol results in the adenosine diphosphate (ADP)-ribosylation of monomeric actin and in the disorganization of the cytoskeleton, with the formation of microtubule-based cell protrusions and an increased adhesion of bacteria to intestinal cells [123,124]. Since CDT targets a broader array of cells in vivo, higher rates of CDI recurrences and lower clinical cure rates are reported for patients infected with strains producing CDT compared to those infected by historical strains [125]. In addition to CDT production, it has been demonstrated that highly virulent *C. difficile* strains produce a TcdB exhibiting broader tropism and cytotoxicity activity compared to the TcdB of historical strains, leading to more rapid cell entry and the increased activity of the toxin [126].

## 8. Gut Microbiota Changes in Patients with *C. difficile* Infection

In general, a decrease in species richness and microbial diversity has been observed in patients with CDI [35]. In addition, a decrease in butyrate-producing *Ruminococcaceae*, *Lachnospiraceae*, and *Clostridia* clusters IV and XIVa bacteria has been observed [127,128,129] (Figure 2B).

*Ruminococcaceae* and *Lachnospiraceae* from the Firmicutes phylum metabolize primary bile acids into secondary bile acids; therefore, if the abundances of these bacterial families decrease, there is also a decrease in secondary bile acids and a consequent decrease in the inhibition of *C. difficile* germination, facilitating the development of a CDI. In particular, a significant reduction in *Lachnospira*, *Odoribacter*, *Coprococcus*, and *Anaerostipes*, as well as in *Prevotella* and *Faecalibacterium*, has been reported as an important finding in patients with CDIs [130,131,132,133]. Interestingly, the results obtained in different studies support a depletion of the genera producing butyric acid not only in the case of severe CDIs, but also with mild disease or *C. difficile* colonization [131,133]. Furthermore, Antharam et al. have found a decrease in the bacteria producing butyric acid, compared to healthy individuals, not only in the intestinal microbial community of patients with CDI but also in those of patients with non-*C. difficile* AAD [134]. 

An increase in the phyla Firmicutes, Proteobacteria, and Actinobacteria and members of the *Gammaproteobacteria*, *Lactobacillaceae,* and *Enterococcaceae* families has been reported in the gut microbiota of people suffering from a CDI compared to healthy people, in addition to a decrease in the relative abundance of Bacteroidetes [134,135,136,137,138] (Figure 2B). However, regarding the phylum Bacteroidetes, data from the literature are not concordant, since some studies show a general decrease in abundance, while others show an increase [135]. Several studies have also reported an increase in the genera *Parabacteroides*, *Enterococcus*, *Klebsiella*, and *Akkermansia* in the microbiota of patients with CDIs, a phenomenon resulting from the competition for a reduced ecological niche [88,127,133]. In addition, Han et al. suggest the depletion of some species, such as *Phascolarctobacterium* spp., and genera, such as *Lachnospira*, *Butyricimonas*, *Catenibacterium*, *Paraprevotella*, *Odoribacter* and *Anaerostipes*, as a signature change in CDIs [133] (Figure 2B).

Interestingly, there is evidence that the gut microbiota can modulate a CDI’s severity, and it has been demonstrated that the relative abundance of *Akkermansia*, *Anaerotignum*, *Blautia*, *Enterocloster*, *Lactonifactor*, and *Monoglobus* is associated with a decrease in *C. difficile* toxin production, a low histopathologic scoring of the cecal tissue, and low mortality in a mouse model [139].

The study of Chen et al. showed that some pathogens are strongly associated with CDIs, such as *Enterocloster bolteae* and *Ruminoccocus gnavus* [43]. *E. bolteae* is an opportunistic pathogen linked to the expression of inflammatory genes; similarly, *R. gnavus* produces glucorhamnan, a potent inflammatory polysaccharide [140,141]. Opportunistic pathogens, such as *Bacteroides*, *Enterococcus*, and *Klebsiella*, as well as *Prevotellaceae*, *Eggerthella,* and *Helicobacter*, have been commonly detected in CDI cases with a higher histopathologic score and worse outcomes [139,142].

Profound alterations were found in patients with recurrent CDI who had received multiple antibiotic treatments [143]. The available literature indicates that the gut microbiota of patients with recurrent CDI shows a reduction in the diversity and richness of its components, as observed in patients with a primary CDI, although some taxa can display different trends in patients with recurrent CDI compared to healthy individuals and patients with a primary CDI. In recent studies analyzing the fecal samples of patients with recurrent CDI, a relative reduction in Bacteroidetes and Firmicutes and an increase in Proteobacteria’s abundance compared to healthy individuals have been observed [143,144]. Khanna et al. have reported that patients with recurrent CDI had a statistically significant increase in *Enterobacteriaceae*, *Lachnospiraceae*, *Streptococci*, *Parabacteroides,* and *Veillonella* levels compared to patients with CDI [144]. In addition, Gazzola et al. have reported a strong reduction in gut commensal *Parabacteroides* in patients with recurrences compared to patients with primary CDI [145]; however, this result disagrees with that of Khanna et al., who suggest that the increase in Parabacteroides is a predictor of recurrence [144].

Weingarden et al. have reported that two primary bile acids, cholic acid and chenodoxycholic acid, are significantly elevated in patients with a recurrent CDI [146]. A more recent investigation confirmed these results, reporting an increase in primary bile acids in the stools of patients with recurrences compared to healthy controls and patients with a primary CDI, which are associated with a reduced abundance of species capable of metabolizing bile acids, such as *Collinsella*, and an increase in the relative abundance of *Enterobacteriaceae* and *Lactobacillus* [147]. 

The data available indicate that there are significant differences in the gut microbiota composition of CDI patients compared to those of carriers and healthy individuals, suggesting that the presence or absence of certain bacteria taxa are often more impactful in determining the development of a CDI than diversity or species richness alone.

## 9. Highly Virulent *C. difficile* Types

The epidemiology of CDIs has been changing in recent decades. Until the end of the 20th century, CDI was considered a complication of antimicrobial exposure and occurred mainly in the hospital environment [148]. Since then, CDIs have been more frequently detected in the community and in populations usually not affected by CDI (antibiotic-naïve people, peripartum women, and children) [8]. Also, the incidence of CDI recurrence has risen, sometimes disproportionately compared to CDIs. For example, between 2001 and 2012, the increase in CDI recurrences was four times higher compared to that of primary CDIs diagnosed in the USA [149].

An increase in *C. difficile* diversity has been observed worldwide, with the emergence of more virulent and antibiotic-resistant *C. difficile* types, which are considered the main driver of recent CDI epidemiological changes [7,150].

Starting in the early 2000s, the PCR-ribotype (RT) 027 has been reported as cause of many outbreaks in the hospitals of North America and Europe [151,152,153]. Its acquisition of a resistance to fluoroquinolones has been identified as the main cause of the global diffusion of the RT027 due to the selective pressure exerted by the widespread use of these antibiotics in healthcare settings. A whole-genome sequence (WGS) analysis has demonstrated that two genetically distinct lineages of RT 027 independently acquired resistance to fluoroquinolones and successively spread: one in the USA, South Korea, and Switzerland and the other in the UK, Europe, and Australia [154]. Due to their high rate of sporulation, increased expression of TcdA and TcdB, production of the binary toxin CDT, and resistance to antibiotics, RT 027 strains have been associated with an overall increase in CDIs’ incidence, severity, rates of recurrence, and mortality [121,155,156,157,158].

RT 078 emerged shortly after RT027 as a cause of infection in hospitals and in the community, with rates of severe diarrhea and an attributable mortality similar to those of RT 027 [159]. *C. difficile* RT 078 is able to infect/colonize livestock and its zoonotic potential is supported by a recent genomic analysis that demonstrated the spread of RT 078 clones across multiple continents, often without any association with the healthcare system, suggesting a zoonotic/anthroponotic long-range dissemination in the community [160]. 

New RTs can evolve from each other, constituting different *C. difficile* lineages [161]. In fact, recent types, such as RT 176 and RT 244, which have been found to be genetically related to RT 027 and have the same characteristics of virulence, constitute the RT 027 lineage [162,163]. Similarly, several types have been identified as related to RT 078, such as RT 126, RT 127, RT 033, and RT 288, constituting the RT 078 lineage [160].

The emergence of highly virulent types has led to the need to understand the possible impact of these new types on the gut microbiota community in order to develop more precise and sustainable approaches to CDIs’ prevention and treatment.

## 10. Impact of Highly Virulent *C. difficile* Types on Gut Microbiota

The recent increase in CDI cases in the community, without previous antibiotic treatment [164], suggests that *C. difficile* might also colonize the gut in the presence of non-dysbiotic microbiota and that this pathogen might be able to affect the gut microbiota’s composition. This hypothesis has been supported by the results obtained in a recent study, which indicate that *C. difficile* is able to alter the level of gene transcription and the metabolomic profile of the gut microbiota, causing a restructuring of the landscape of nutrients in the intestine and promoting persistent infection [165].

Robinson et al., using fecal minibioreactor arrays, have demonstrated that *C. difficile* colonization significantly perturbs the intestinal microbiota’s composition, particularly when certain *C. difficile* types are used [166]. In fact, it has been observed that RT 027 strains have an ecological advantage not only over other *C. difficile* types but also over the gut microbiota’s components due to their capability to exploit the limited nutrients available. A recent study has demonstrated that toxin-producing *C. difficile* colonization causes an alteration in the nutrient pool and that the inflammation induced by *C. difficile* RT 0127 toxin activity leads to alterations in the components of the gut microbial community, with a suppression of members from the family *Bacteroidaceae* and the increased growth of *C. difficile* [14].

Skraban et al. have analyzed the presence of bacterial, fungal, and archaeal microbiota in fecal samples from healthy individuals and patients with a request for *C. difficile* testing, considering the presence or absence of *C. difficile* RT 027 or non-RT 027 in this last group [167]. The results indicate an association between RT 027 strains and low levels of microbiota diversity, suggesting that this type of CDI is potentially capable of affecting the composition of the gut microbiota and, therefore, increasing its own colonization potential.

Interestingly, Horvat et al. have evaluated the effects of *C. difficile* on the gut microbiota using six different *C. difficile* strains belonging to RT 027 and to RT 014/020 for the co-cultivation of fecal microbiota in an *in vitro* model [168]. A general decrease in the microbial diversity of the fecal microbiota has been observed with both *C. difficile* types. Although the changes in the microbiota’s composition due to RT 027 and RT 014/020 are different, many of these alterations occur within the phylum Firmicutes, with a general decrease in commensals with potential protection functions (i.e., *Lactobacillus*, *Clostridium*_XIVa) and an increase in opportunistic pathogens (i.e., *Enterococcus*). Furthermore, fecal microbiota co-cultivated with other bacteria (*Escherichia coli*, *Salmonella Enteritidis,* or *Staphylococcus epidermidis*) show different patterns of alterations, supporting the hypothesis that *C. difficile* effects are specific. In another investigation, the same authors analyzed the interactions between different *C. difficile* RTs and two types of fecal microbiota (healthy and dysbiotic) using an *in vitro* batch model [169]. The results have indicated that, although strains belonging to all the RTs tested show a higher frequency of sporulation in the presence of dysbiotic fecal microbiota, the growth is strain-dependent. 

The results obtained by several authors indicate a decrease in the richness and diversity of the fecal microbiota of both adults and children when co-cultured with different *C. difficile* types, with a decrement in the butyrate-producing members of *Ruminococcaceae* and *Lachnospiraceae* and an increase in Proteobacteria members [167,169,170,171,172]. In addition, important metabolic alterations were reported in the microbiota of children when co-cultured with different *C. difficile* types. In particular, higher concentrations of acetic acid, butyric acid, phenylacetic acid, p-hydroxyphenylacetic acid, and tyramine were detected when the fecal microbiota were co-cultured with *C. difficile* RT 027 and RT 176, which are associated with a great perturbation in the microbiota’s composition and *C. difficile* overgrowth [170].

All these data suggest that alterations in the gut microbiota of patients with CDI could be directly influenced and maintained by *C. difficile*, with effects that differentiate between the different types.

## 11. Microbial Interventions to Restore Gut Microbiota

**Figure 3:**
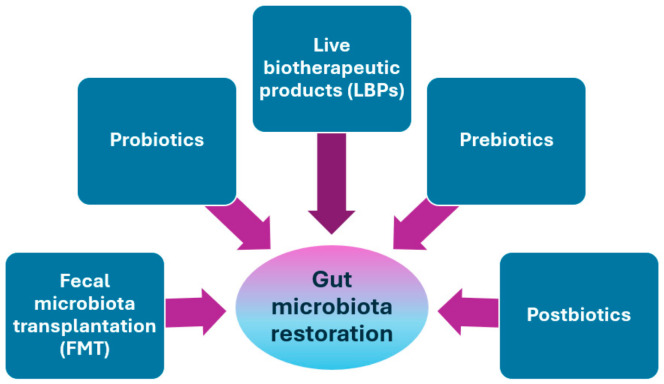
Treatments for gut microbiota restoration in patients with CDI.

Restoring the gut microbiota to a healthy state, or eubiosis, is an important goal for therapeutic and protective approaches to CDI. Microbial interventions can be divided into fecal microbiota transplantation (FMT), the use of probiotics, live biotherapeutic products (LBPs), prebiotics, and postbiotics [20,173] (Figure 3).

### 11.1. Fecal Microbiota Transplantation (FMT)

The efficacy of the gut microbiota’s restoration in the treatment of CDI has been demonstrated by the high percentage of clinical cure obtained with fecal microbiota transplantations (FMTs). An FMT consists of restoring the microbial diversity and the metabolic landscape of the gut microbiota of a patient with CDI through the delivery of feces from a healthy subject [174].

A first study, performed in 2013 by van Nood et al. and including patients with CDI treated with oral vancomycin followed by bowel lavage and a nasoduodenal infusion of feces solution from a donor; or with vancomycin combined with bowel lavage; or only with vancomycin, demonstrated that 81% of patients who received donor feces had a complete resolution of their symptoms after the first treatment [175].

A subsequent study demonstrated that employing an FMT via colonoscopy yields superior results in treating recurrent CDI compared to a nasoduodenal infusion, with a 90% of success rate versus a vancomycin regimen [176]. Although an FMT colonoscopy is commonly used, oral FMT capsules show an efficacy in treating recurrences of CDI that is comparable to colonoscopy, regardless of whether frozen or lyophilized stool is used for capsule preparation [177]. The use of frozen or lyophilized stool for FMT is supported by a recent study, which showed that the marginal decrease in relative efficacy from fresh FMT (93% efficacy) to frozen FMT (88% efficacy) and lyophilized FMT (83% efficacy) can be outweighed by the accessibility and ease of use of frozen or lyophilized preparations [178]. In addition, colonoscopy or oral capsules as administration routes for FMTs have an efficacy that overcomes enemas or nasogastric tubes [179].

An FMT is considered generally safe since its adverse events (loose stool, abdominal discomfort, or diarrhea), which occur in up to 50% of patients, are transient or self-limiting [180,181].

More recently, a new methodology known as washed microbiota transplantation (WMT), in which the stool specimen from a donor is automatically filtered and washed, has reduced the incidence rate of fever due to bacterial translocation across the ulcerated intestinal mucosa from 19.4% (manually prepared FMT) to 2.7% (WMT) [182]. In a multicenter study performed in China, patients affected by CDI were treated with WMT by a colonic transendoscopic enteral tube introduced via colonoscopy and secured in the colon with an endoclip that allowed for repeated procedures, obtaining a clinical cure rate of 90.7% [183].

Even though FMT demonstrates superiority in the treatment of recurrent CDI, there are still critical aspects that have limited its diffusion, such as a lack of dedicated structures, difficulties in the recruitment/selection/screening of donors, and the safety of patients. Internationally, panels of experts are involved in its methodological implementation, the standardization of stool banks, and quality assurance to guarantee accessibility to FMT worldwide [184,185].

#### Gut Microbiota Changes in FMT Patients

Unlike vancomycin and fidaxomicin monotherapies, which inhibit the immediate growth of *C. difficile*, FMT induces a durable treatment response, sustained through several mechanisms, including *C. difficile*’s competition with the FMT-delivered microbiota, the restoration of secondary bile acid metabolism, the stimulation of the mucosal immune system, and the repair of the gut barrier [186,187].

Several studies have observed that the composition of the fecal samples of pre-FMT patients shows a reduced relative abundance of bacterial families from the Bacteroidetes and Firmicutes phyla, such as *Bacteroidaceae*, *Clostridiaceae*, *Eubacteriaceae*, *Lachnospiraceae*, *Prevotellaceae*, and *Ruminococcaceae*, and an increase in the relative abundance of the Proteobacteria phylum, in particular of the *Gammaproteobacteria*, which are restored after FMT [188,189,190,191,192].

**Figure 4:**
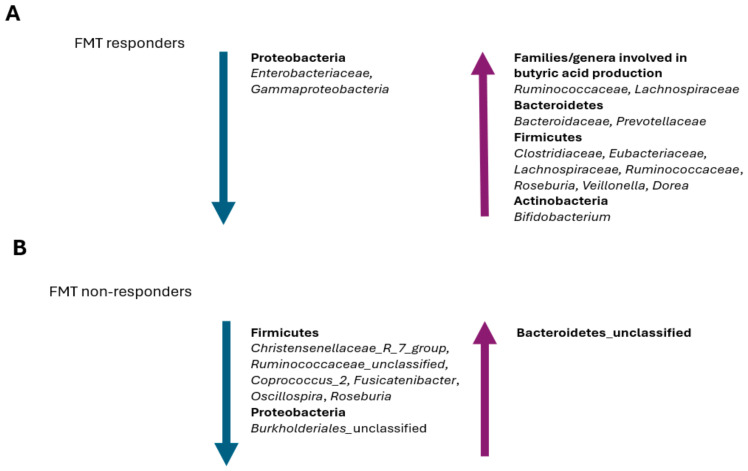
Prevalent changes observed in the gut microbiota of patients with recurrent CDI that (**A**) positively respond to FMT and (**B**) negatively respond to FMT.

A recent study investigated the gut microbiota changes that occurred in patients treated with vancomycin followed by FMT to compare the differences between responders and non-responders to this treatment [193]. Although no significant differences have been observed immediately after an FMT, after one week, responders have shown a gut microbiota similar to healthy donors, depleted of *Enterobacteriaceae* and enriched in *Lachnospiraceae* and *Ruminococcaceae* (Figure 4A).

In a recent study by Tian et al., a reduction in the abundance of butyrate-producing bacteria of the Firmicutes phylum, including *Christensenellaceae*_R_7_group, *Ruminococcaceae*_unclassified, *Coprococcus*_2, *Fusicatenibacter*, *Oscillospira*, and *Roseburia*, was observed in the FMT non-responders [194] (Figure 4B). In fact, butyrate stabilizes the expression level of hypoxia-inducible factor-1 (HIF1) and can improve the intestinal barrier, prevent bacterial translocation, and reduce the local inflammatory response [195,196]. Thian et al. have also observed that both patients prior to an FMT and FMT non-responders show a reduction in their relative abundance of *Burkholderiales*_unclassified, *Coprococcus*_2, and *Oscillospira*, while an enrichment in Bacteroidetes_unclassified has been observed in FMT non-responders [194] (Figure 4A,B). Since *Burkholderia* has an important negative impact on CDI pathogenesis by inhibiting the Rho-glucosylation activity of *C. difficile* TcdB, its reduction or depletion led to a higher probability of treatment failure in FMT patients [197]. Similarly, *C. difficile* metabolic activities require the consumption of the succinic acid that is produced by *Bacteroides* [103]; therefore, an enrichment in Bacteroidetes_unclassified in FMT non-responders could potentially favor the recurrence of CDI (Figure 4B).

The recovery of both Roseburia and Veillonella, which are able to inhibit *C. difficile*, is usually correlated with a positive response to an FMT treatment and CDI recurrence resistance [198,199] (Figure 4A). In fact, a higher relative abundance of certain bacteria, including *Veillonella*, in the gut microbiota of the recipient prior to an FMT treatment seems to favor a positive response to the treatment itself [194]. Interestingly, the relative abundance of *Veillonella* gradually reduces with age and its depletion is probably related to higher rates of CDI and CDI recurrences in these patients [200]. Therefore, a *Veillonella* transplant should not be ignored in the FMT treatment of this patient population.

*Bifidobacterium*, *Dorea*, and *Ruminococcaceae* have mucinophilic properties similar to *Bacteroides* and *C. difficile* [194]; therefore, it is possible that, in FMT responders, these bacteria can promote the displacement of *C. difficile* through niche competition, preventing CDI recurrences. In general, *Oscillospira* is less abundant in the gut microbiota of patients with gastrointestinal inflammatory diseases, such as inflammatory bowel disease [191,201]. The depletion of *Oscillopora* in the gut microbiota of donors, as well as the absence of *Burkholderiales*_unclassified and *Coprococcus*_2, may be an important reason for the low effectiveness of FMT, since successful colonization of the FMT recipient’s intestine is largely due to the phylogeny of the gut microorganisms of donors [202].

Aggarwala et al., applying the Strainer algorithm, have tracked sequenced bacterial strains in metagenomic sequencing datasets from donors and FMT patients up to 5 years post-FMT [192]. The results obtained have demonstrated that >70% of strains from donors and <25% of strains from non-relapsing FMT recipients have been retained 5 years post-FMT. These data suggest that FMT represents a durable therapeutic treatment that determines a semipermanent alteration of the recipient’s gut microbiota after a single administration. The authors also observed that the engraftment efficacy of FMT donors’ microbiota differed by bacterial species [192]. Some species were able to stably engraft in the gut of recipients, such as those belonging to *Bacteroidales*, which were always engrafted. In fact, *Bacteroidales* have a higher transmission efficiency compared to other bacteria [203]. Contrastingly, Firmicutes have reduced and more difficult intestinal colonization, with complex adaptive activity that occurs at the expense of spore-forming activity, favoring intestinal colonization by non-spore-forming Firmicutes, such as *Coprococcus*_2 and *Oscillospira*, whose depletion is associated with poor prognosis in FMT patients [204,205].

### 11.2. Probiotics

Probiotics are defined as “live microorganisms that, when administered in adequate amounts, confer a health benefit on the host” [206]. Probiotics can be used to prevent the onset of dysbiosis or as therapeutic agents for an ongoing condition of dysbiosis (Figure 3).

Probiotics belong to bacterial species that are normal components of the human gut microbiota, designated as “Generally Regarded As Safe (GRAS)”, which are able to resist the gastric environment (bile and pancreatic secretions) and to be active and vital for a reasonable period in the intestine [207]. The ability to hydrolyze bile salts is often included as a criterion for the selection of probiotic strains.

The most commonly used probiotic microorganisms include *Lactic acid* bacteria, *Bifidobacteria*, *Enterococci*, *Probionibacteria*, *Bacillus* spp., the *Escherichia coli* strain Nissle 1917, and the yeast *Saccharomyces boulardii* [208,209]. Most of these probiotics act through competitive exclusion, modulating the gut microbiota and restoring a healthy intestinal microbial community, with a reduction in cholesterolemia, inflammation, diarrhea episodes, bowel discomfort, and a suppression of colon cancer and allergic states.

Several studies have evaluated the use of antibiotics in combination with probiotics for recurrent CDIs. In one randomized controlled trial, *S. boulardii* was used in combination with standard antibiotics for CDIs in patients with primary or recurrent CDI, showing that those receiving *S. boulardii* had fewer recurrences in comparison to those treated with a placebo [210]. In another study, patients with CDI recurrences were treated with high-dose vancomycin (2 g/d), low-dose vancomycin (500 mg/d), or metronidazole (1 g/d), in combination with *S. boulardii* (1 g/d), with a reduction in recurrences in the group treated with high-dose vancomycin followed by *S. boulardii* [211].

Wullt et al. have observed that patients with recurrent CDI treated with metronidazole for 10 days, followed by a dose of *Lactobacillus plantarum*, show fewer recurrences compared to those treated with a placebo, supporting *L. plantarum* as a probiotic that could be useful in preventing CDI, as also observed in other studies [212,213,214]. Also, a probiotic mixture including *Lactobacillus acidophilus* NCFM, *Lactobacillus paracasei* Lpc-37, *Bifidobacterium lactis* Bi-07, and *B. lactis* Bl-04 has been administered to patients with mild to moderate CDI, significantly improving infection duration and diarrhea severity compared to a placebo [215].

Recent studies have reported an association between the use of *Lacticaseibacillus rhamnosus* as a probiotic and increased butyrate production [216,217,218]. Furthermore, other studies have investigated *C. butryicum*, which promotes resistance to *C. difficile* infection, as a possible probiotic for CDI [56,57,58,59].

Although several studies have investigated the effects of probiotics on *C. difficile* colonization, growth, or toxin production, considering probiotics as an adjunctive therapy for CDI in combination with antibiotic treatment [219,220,221,222,223,224], their role in preventing CDI is still unclear. For these reasons, the current guidelines do not recommend the use of probiotics for primary CDI or CDI recurrence prevention [225,226].

Finally, the potential adverse events seen with probiotics remain crucial. In this respect, an association between the use of probiotics and increased mortality has been observed in a randomized, double-blind, placebo-controlled trial investigating the use of probiotics to prevent predicted severe pancreatitis [227]. Although there is evidence that probiotics can prevent CDI and that better therapeutic outcomes are obtained using probiotics, it is still not possible to know if these various probiotics are universally safe for every subject.

### 11.3. Live Biotherapeutic Products (LBPs)

Ready-to-prescribe formulations of live biotherapeutic products (LBPs), which are medicinal products containing live microorganisms (bacteria or yeasts) for human use, are considered a simplified option for daily CDI clinical practice (Figure 3).

In 2022, the US Food and Drug Administration (FDA) approved RBX2660, marketed as “REBYOTA™, for preventing CDI recurrences in adults who have undergone standard antibiotic treatment. RBX2660 consists of a consortium of live microbes from human feces, delivered as an enema. The efficacy of RBX2660 has been demonstrated in a randomized, double-blind, placebo-controlled phase III trial involving 267 adult patients with one or more CDI recurrences, with a success rate (defined as the absence of *C. difficile*-associated diarrhea within 8 weeks of the study treatment) of 70.6%, compared to 57.5% for the placebo group [228]. A metabolomic analysis has demonstrated a shift from a majority of primary bile acids to predominantly secondary bile acids when the RBX2660 is used [229].

The advantages of RBX2660 are its efficacy in reducing the recurrence of CDI, its cost-effectiveness, and safety profile, while the disadvantages include side effects (mild gastrointestinal complaints), its storage, and the need for specialized handling. Furthermore, the mode of administration may pose logistical challenges for some facilities and patients [230]. 

In 2023, the FDA approved SER-109 (VOWST™), which consists of purified encapsulated Firmicute spores that are administered orally over three consecutive days. Its efficacy has been demonstrated in a randomized, double-blind, placebo-controlled phase III trial involving 182 patients with three or more CDI recurrences, with a SER-109 success rate of 88% compared to 60% with the placebo [231]. In the study, mild to moderate gastrointestinal adverse events were observed after the SER-109 treatment. Interestingly, SER-109 species have been detected in treated patients after a week, and they have been associated with bile-acid profiles known to inhibit the germination of *C. difficile* spores.

Ongoing clinical trials are investigating other LBPs based on non-pathogenic strains, such as VE303 and NTCD-M3, which can be orally administered [232,233]. VE303, from Vedanta Biosciences, includes eight strains of non-pathogenic commensal *Clostridia* that are grown in clonal stool banks and encapsulated for oral delivery. The results from a phase II, randomized, double-blind, placebo-controlled, dose-ranging study involving adult patients with one or more recurrences of CDI and patients with primary CDI at risk of recurrences have demonstrated that high-dose VE303 prevents CDI recurrences in both groups of patients compared to the placebo (13.8% vs. 45.5%, *p* = 0.006) [234]. NTCD-M3 is a novel live LBP that consists of spores of the nontoxigenic *C. difficile* strain M3. A phase II clinical trial involving 173 patients with primary CDI or recurrent CDI has demonstrated that NTCD-M3 is able to prevent CDI recurrences when administered shortly after the antibiotic treatment of a primary CDI, with 11% of CDI recurrences in the NTCD-M3 group of patients compared to 30% in the placebo group [235].

### 11.4. Prebiotics

The current prebiotic concept refers to non-digestible compounds that, through their metabolization by microorganisms colonizing the gut, modulate the composition and the activity of the gut microbiota, conferring beneficial effects to the host [234] (Figure 3).

Prebiotics, which can be in natural or synthesized forms (inulin, oligosaccharides, lactulose, pyrodextrins, dietary fibers, and resistant starches, etc.) [234], are used as a complementary therapy for the treatment of various diseases, such as inflammatory bowel disease, Parkinson’s disease, or obesity [235].

The combination of a prebiotic with a probiotic to favor and enhance the colonization and growth of certain microorganisms is known as a “synbiotic” [235]. Rätsep et al. have demonstrated that the combination of xylitol and *L. plantarum Inducia* is able to suppress the germination of *C. difficile* spores and their outgrowth into vegetative forms in hamsters, reducing the colonization of the gut by this pathogen [236]. This therapeutic approach includes the use of the synbiotic during antimicrobial therapy for the prevention of CDI and to reduce recurrences. Valdés-Varela et al. have evaluated the *in vitro* inhibitory potential of four bifidobacterial strains, co-cultured with different prebiotics as carbon sources, on *C. difficile* growth and on the toxicity of *C. difficile* culture supernatants toward human intestinal epithelial cells HT29 [237]. Their co-culture with *B. longum* IPLA20022 or *B. breve* IPLA20006 in the presence of two commercial short-chain fructooligosaccharides (scFOSs) has been demonstrated to significantly reduce the growth of *C. difficile* and the toxicity of *C. difficile* supernatants. These results suggest that the optimal prebiotic substrate is probably strain-specific, so further investigations will be necessary to determine the best prebiotic substrate for different probiotic strains and the effects of synbiotics on the human intestinal microbiota.

### 11.5. Postbiotics

Salminen et al. defined a postbiotic as a “preparation of inanimate microorganisms and/or their components that confers a health benefit to the host” [238]. Postbiotics can act indirectly, modulating the microbiome’s composition, or directly, regulating the immune system [239] (Figure 3).

Currently, postbiotics derived from the industrial fermentation of the *Lactobacillus* or *Bifidobacterium* bacterial genera or from yeasts, such as *Saccharomyces cerevisiae*, are commonly metabolites or polysaccharides with antioxidant, anti-inflammatory, or immunomodulatory properties [240,241]. Novel postbiotics include whole inactivated bacteria cells, such as pasteurized *Akkermansia muciniphila*, which can improve glycemia and obesity rates [242]. 

Postbiotics represent a new option for preventing/treating CDIs. In fact, probiotics from various lactic acid bacteria, when co-cultured with toxigenic *C. difficile*, can reduce the cytotoxic effects of toxins on various cell lines, as well as the level of IL-8 and tumor necrosis factor α (TNF-α) released by intestinal epithelial cells when infected by *C. difficile* [242,243]. The lyophilized cell-free supernatant from bacteriocin-like-producing *Enterococcus faecalis* has been demonstrated to act as a postbiotic, with antibacterial activity against *C. difficile* and the capacity to inhibit the germination of spores [242].

Probiotics research is a rapidly evolving field due to their safety, especially for vulnerable patients, their longer shelf life, and their possibility of being used in topical formulations [243,244].

## 12. Conclusions

A *C. difficile infection* (CDI) is typically the result of perturbations in the gut microbiota’s composition, mainly due to the use of antibiotics. The gut microbiota has a key role in resisting *C. difficile*; therefore, alterations in its microbial structure create conditions conducive to *C. difficile*’s colonization and infection. Data on the gut microbiota’s composition indicate a decrease in species richness/diversity in both asymptomatic *C. difficile* carriers and patients with CDI. However, the presence or absence of certain bacteria taxa often appears more impactful in *C. difficile* colonization or in the development of a CDI than their diversity or species richness alone. Furthermore, recent data support the hypothesis that the gut microbiota can modulate a CDI’s severity, but also that *C. difficile*, particularly highly virulent types, such as RT 027, can directly influence and maintain alterations in the gut microbiota of patients with CDIs.

Accumulating data suggest that gut microbiota manipulations are an important therapeutic strategy for the prevention and treatment of CDIs. A variety of microbiota-based approaches have been developed, with some already adopted in clinical practice (i.e., FMT) and some still under investigation. However, all these approaches have still face challenges related to their standardization and safety concerns that deserve further investigation.

The increasing volume of information and the development of precise and in-depth datasets on the gut microbiota composition profiles of patients and healthy individuals will allow us to predict responses to CDI treatment and develop personalized microbiota therapies, which could be decisive for the appropriate prevention and management of CDIs in the near future.
